# Optimization of InGaAs/InAlAs Avalanche Photodiodes

**DOI:** 10.1186/s11671-016-1815-9

**Published:** 2017-01-13

**Authors:** Jun Chen, Zhengyu Zhang, Min Zhu, Jintong Xu, Xiangyang Li

**Affiliations:** 1 School of Electronic and Information Engineering, Soochow University, Suzhou, 215006 China; 2 Key Laboratory of Infrared Imaging Materials and Detectors, Shanghai Institute of Technical Physics, Chinese Academy of Sciences, Shanghai, 200083 China

**Keywords:** Avalanche photodiodes (APDs), Punchthrough voltage, Breakdown voltage, Simulation

## Abstract

In this paper, we report a two-dimensional (2D) simulation for InGaAs/InAlAs separate absorption, grading, charge, and multiplication avalanche photodiodes (SAGCM APDs) and study the effect of the charge layer and multiplication layer on the operating voltage ranges of APD. We find that with the increase of the thicknesses as well as the doping concentrations of the charge layer and the multiplication layer, the punchthrough voltage increases; with the increase of the doping concentrations of two layers and the thickness of the charge layer, the breakdown voltage decreases; with the increase of the thickness of the multiplication layer, the breakdown voltage first rapidly declines and then slightly rises.

## Background

Focal plane array (FPA) based on In_0.53_Ga_0.47_As (referred as InGaAs hereafter) has a huge market and wide application prospect, and it is widely used in military field [1]. For narrow band gap materials like InGaAs, high tunneling current limits their usefulness. Separating the absorption and multiplication layer can overcome this disadvantage [2]. InGaAs is often used to absorb light at a wavelength of 1.55 μm, while for the multiplication layer, In_0.52_Al_0.48_As (referred as InAlAs hereafter) is a good multiplication layer material [3]. InAlAs has been demonstrated to be a good electron multiplication material for InGaAs separate absorption and multiplication avalanche photodiodes (SAM APDs) because of its low electron impact ionization threshold energy of 1.9–2.2 eV, high ionization coefficient ratio of electron to hole than that of hole to electron in InP, and small excess noise factor [4, 5].

For separate absorption, grading, charge, and multiplication avalanche photodiodes (SAGCM APDs), the key issue is to adjust the electric field distribution in the device by changing the thickness and doping concentration of the charge layer and the multiplication layer. Provided that the electric field is sufficiently large in the multiplication region, the carriers will undergo avalanche multiplication, and the device behaves as an avalanche photodetectors (APD) as desired [6]. The SAGCM structure allows independent control of the parameters of the charge layer and the multiplication layer (thickness and the doping concentration). In this paper, we study the effect of the charge layer and multiplication layer on the operating voltage ranges of APD and analyze the results theoretically from the internal electric field distribution.

## Methods

**Fig. 1:**
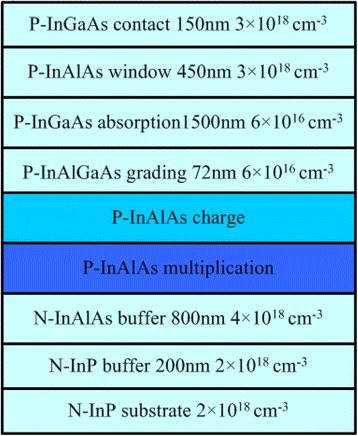
Structure of InAlAs/InGaAs APD

Figure 1 shows the schematic cross-section of a top-illuminated SAGCM InGaAs/InAlAs APD with 400 μm^2^ mesa structure. From the top to the bottom, these layers are sequentially named as contact layer, window layer, absorption layer, grading layer, charge layer, multiplication layer, InAlAs buffer layer, InP buffer layer, and InP substrate. The device structure in our simulation is the same as the experimental device reported in Ref. [7].

The steady-state two-dimensional (2D) numerical simulations are performed for the top-illuminated SAGCM InGaAs/InAlAs APD by using Silvaco TCAD [8]. The Shockley–Read–Hall (SRH), auger, band-to-band tunneling, and trap-assisted tunneling models are used in our simulation. The generation rate *G*_bbt_ of band-to-band tunnel is described in Eqs. (1) and (2) [8].

1 $$ {G}_{\mathrm{bbt}}=A\cdot E\cdot \exp \left(-\frac{B}{E}\right) $$

2 $$ A=-\frac{q^2\sqrt{2{m}_e^{\ast }}}{4{\pi}^3{h}^2\sqrt{E_g}}\kern2.5em B=\frac{\pi \sqrt{m_e^{\ast }/2}{E}_g^{3/2}}{2q\hslash } $$

The *A* and *B* are the characterization parameters; *E* is the magnitude of electric field, and *E*_g_ is the band gap energy level. The generation rate *R*_tat_ in trap-assisted tunneling process is given in Eqs. (3)–(5) [8–11].

3 $$ {R}_{\mathrm{t}\mathrm{at}}=\frac{pn-{n}_{\mathrm{i}}^2}{\frac{\tau_p}{1+{\varGamma}_p}\left[n+{n}_{\mathrm{i}}\cdot \exp \left(\frac{E_{\mathrm{t}}-{E}_{\mathrm{i}}}{kT}\right)\right]+\frac{\tau_n}{1+{\varGamma}_n}\left[p+{n}_{\mathrm{i}}\cdot \exp \left(\frac{E_{\mathrm{i}}-{E}_{\mathrm{t}}}{kT}\right)\right]} $$

4 $$ {\varGamma}_{n,p}=\frac{\varDelta {E}_{n,p}}{kT}{\displaystyle {\int}_0^1 \exp \Big(}\frac{\varDelta {E}_{n,p}}{kT}u-{K}_{n,p}{u}^{3/2}\Big)du $$

5 $$ {K}_{n,p}=\frac{4}{3}\frac{\sqrt{2{m}_{\mathrm{trap}}{\left(\varDelta {E}_{n,p}\right)}^3}}{3qh\left|E\right|} $$

where *τ*_*n*_ (*τ*_*p*_) is the electron (hole) lifetime due to the SRH recombination. *E*_t_ is the trap level, and *N*_t_ is the trap concentration. *E*_i_ is the intrinsic Fermi level, and *n*_i_ is the intrinsic carrier concentration. *Γ*_*n*_ (*Γ*_*p*_) is the enhancement factor and includes the effects of field-assisted tunneling on the emission of electrons (holes) from a trap, *ΔE*_*n*_ (*ΔE*_*p*_) is the energy range where tunneling can occur for electrons (holes), *u* is the integration variable, and *m*_trap_ is the effective mass used for carrier tunneling. The effect of carrier avalanche is accounted for by the impact ionization model, which has the following forms:

6 $$ {\mathrm{G}}^{ava}={\alpha}_nn{v}_n+{\alpha}_pp{v}_p $$

Where *α*_*n*,*p*_ are the electron and hole ionization coefficients, respectively, [8, 12, 13]

7 $$ {\alpha}_{n,p}(F)=\gamma {a}_{n,p}{e}^{-\frac{\gamma {b}_{n,p}}{F}} $$

The parameters above are listed in Table 1.

**Table 1:** Material parameters used for InGaAs/InAlAs APD simulation [6, 8, 16, 17]

Parameters/InAlAs	Units	Electron	Hole
SRH lifetime	s	1 × 10^−6^	1 × 10^−6^
Radioactive coefficient	cm^3^ s^−1^	1.2 × 10^−10^	1.2 × 10^−10^
BBT coefficient *α*	1	2	2
BBT coefficient A	1/V cm s	2.1 × 10^11^	2.2 × 10^6^
BBT coefficient B	V/cm	2.1 × 10^11^	2.2 × 10^6^
Trap level *E*_t_	ev	0.72	
Trap concentrations *N*_t_	cm^−3^	1 × 10^−12^	
*m*_trap_	m_0_	0.03	
Impact coefficient a	cm^−1^	1.3 × 10^7^	3.3 × 10^7^
Impact coefficient b	V/cm	3.5 × 10^6^	4.5 × 10^6^

## Results and Discussion

**Fig. 2:**
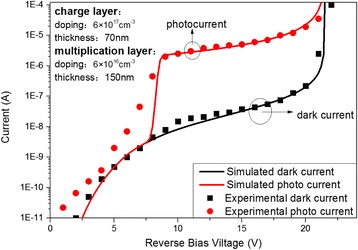
Simulated photocurrent (*red solid line*) and dark current (*black solid line*) as a function of the reverse bias voltage, and experimental photocurrent (*red dotted circle*) and dark current (*black solid line*) of APD from Ref. [7]

Figure 2 presents the simulated and experimental current–voltage (*I–V*) characteristics for the top-illuminated SAGCM InGaAs/InAlAs APD. The simulated results are in good agreement with the experimental data reported in Ref. [7].

**Fig. 3:**
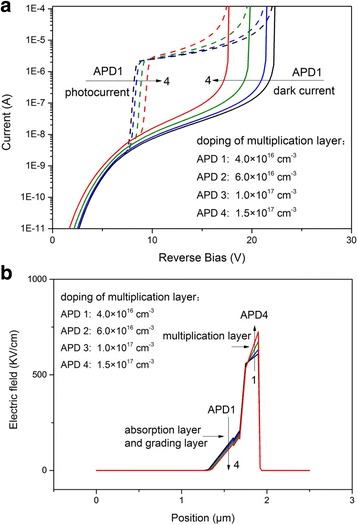
**a** Current–voltage characteristic of avalanche photodiode with different multiplication layer doping. **b** Distribution of electric field, biased at 15 V

The simulated *I*–*V* characteristics at different doping concentrations of the multiplication layer are shown in Fig. 3, the punchthrough voltage (at the unity gain point: the bias where the responsivity of APD reaches ~0.6 A/W) increases monotonically with the increasing of doping concentration (4 × 10^16^ ~ 1.5 × 10^16^ cm^−3^), [14] while the breakdown voltage (dark current ~ 1 × 10^−5^ A) decreases monotonically. With the change of the doping concentration, the electric field in the multiplication layer changes obviously. We analyze the results theoretically following assumptions and simplifications [15]:

1. P^+^–N is an abrupt junction
2. The doping concentrations in the multiplication, charge, grading and absorption layers are uniform
3. If the absorption layer is completely depleted at breakdown voltage, *x*_s_ will be the thickness of the absorption layer

8 $$ {V}_{\mathrm{m}\mathrm{esa}}+{V}_{\mathrm{bi}}=\frac{q{\sigma}_{\mathrm{m}}}{\varepsilon_1{\varepsilon}_0}\left(\frac{x_{\mathrm{m}}}{2}\right)+\frac{q{\sigma}_{\mathrm{c}}}{\varepsilon_1{\varepsilon}_0}\left({x}_{\mathrm{m}}+\frac{x_{\mathrm{c}}}{2}\right)+\frac{q{\sigma}_{\mathrm{g}}}{\varepsilon_2{\varepsilon}_0}\left({x}_{\mathrm{m}}+{x}_{\mathrm{c}}+\frac{x_{\mathrm{g}}}{2}\right) $$

9 $$ \begin{array}{l}{V}_{\mathrm{br}}+{V}_{\mathrm{bi}}={F}_{\mathrm{br}}\left({x}_{\mathrm{m}}+{x}_{\mathrm{c}}+{x}_{\mathrm{g}}+{x}_{\mathrm{s}}\right)-\frac{q{\sigma}_{\mathrm{m}}}{\varepsilon_1{\varepsilon}_0}\left(\frac{x_{\mathrm{m}}}{2}+{x}_{\mathrm{c}}+{x}_{\mathrm{g}}+{x}_{\mathrm{s}}\right)\\ {}-\frac{q{\sigma}_{\mathrm{c}}}{\varepsilon_1{\varepsilon}_0}\left(\frac{x_{\mathrm{c}}}{2}+{x}_{\mathrm{g}}+{x}_{\mathrm{s}}\right)-\frac{q{\sigma}_{\mathrm{g}}}{\varepsilon_2{\varepsilon}_0}\left(\frac{x_{\mathrm{g}}}{2}+{x}_{\mathrm{s}}\right)-\frac{q{\sigma}_{\mathrm{s}}}{\varepsilon_3{\varepsilon}_0}{x}_{\mathrm{s}}\end{array} $$

The *V*_mesa_ is the punchthrough voltage, *V*_bi_ is the zero bias voltage, and *V*_br_ is the breakdown voltage; *x*_m_, *x*_c_, and *x*_g_ are the thickness of the multiplication, charge, and grading layer, respectively; and *σ*_m_, *σ*_c_, *σ*_g_, and *σ*_s_ are the charge density in the multiplication, charge, grading, and absorption layer, respectively, *σ* = *N* ⋅ *x*; and *ε*_0_, *ε*_1_, *ε*_2_, and *ε*_3_ are the dielectric constant of vacuum, InAlAs, InGaAs, InAlGaAs, respectively; *F*_br_ is the electric field in the multiplication layer at breakdown [13]. To get smaller dark currents, larger breakdown voltage, and larger gain factor, the doping of absorption layer is relatively higher [14]. From Eq. (9), when the absorption layer is not completely depleted at breakdown voltage, *x*_s_ is the width of the depletion region of the InGaAs absorption layer.

With the decreasing of doping concentration, the electric field between the absorption layer and the grading layer increases, which makes the electron more easier to punch through the absorption layer and the grading layers, so the punchthrough voltage decreases, owing to the wedge-shaped electric field profile with a high gradient [12]. From Fig. 3, we can see that the doping of the multiplication layer has a great influence on the performance of the device.

**Fig. 4:**
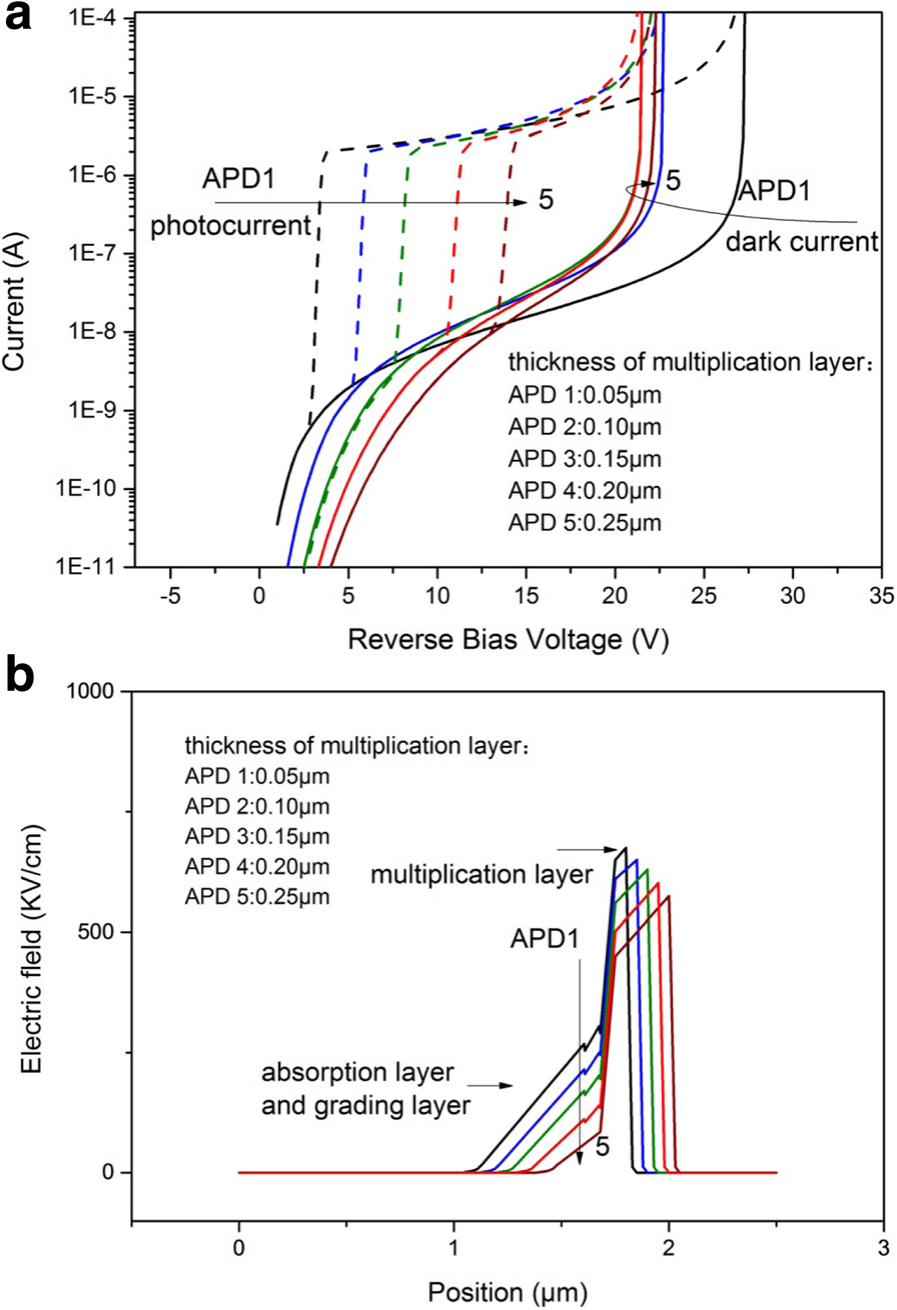
**a** Current–voltage characteristic of avalanche photodiode with different multiplication layer thickenesses. **b** Distribution of electric field, biased at 15 V

**Fig. 5:**
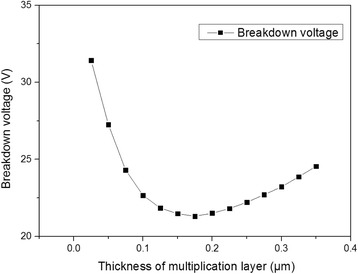
Voltage thickness characteristic of avalanche photodiode

Figure 4 shows the simulated *I*–*V* characteristics with different thicknesses of the multiplication layer (0.05 ~ 0.25 μm). The punchthrough voltage increases with the increasing thickness of multiplication layer, [13] and the breakdown voltage first rapidly declines then slightly rises (Fig. 5). We analyze the results theoretically from Eqs. (8) and (9), and the following equations: [18]

10 $$ {M}_n=\frac{1-1/k}{ \exp \left[-\alpha \left(1-1/k\right){x}_{\mathrm{m}}\right]-1/k} $$

(*M*_*n*_ is the multiplication factor of electron in the multiplication layer)

So, we can get:

11 $$ \frac{\partial {M}_n}{\partial {x}_{\mathrm{m}}}={M}_n^2\left(\alpha +{x}_{\mathrm{m}}\frac{\partial \alpha }{\partial {x}_{\mathrm{m}}}\right) \exp \left[-\alpha \left(1-1/k\right){x}_{\mathrm{m}}\right] $$

12 $$ \frac{\partial \alpha }{\partial {x}_{\mathrm{m}}}=\frac{\partial \alpha }{\partial {E}_{\mathrm{m}}}\frac{\partial {E}_{\mathrm{m}}}{\partial {x}_{\mathrm{m}}} $$

(*E*_m_ is the max electric field intensity in the multiplication layer).

So,

$$ {x}_{\mathrm{m}}>\frac{-\alpha }{\partial \alpha /\partial {x}_{\mathrm{m}}}\Rightarrow \alpha +{x}_{\mathrm{m}}\frac{\partial \alpha }{\partial {x}_{\mathrm{m}}}<0\Rightarrow \frac{\partial {M}_n}{\partial {x}_{\mathrm{m}}}<0, $$

$$ {x}_{\mathrm{m}}<\frac{-\alpha }{\partial \alpha /\partial {x}_{\mathrm{m}}}\Rightarrow \alpha +{x}_{\mathrm{m}}\frac{\partial \alpha }{\partial {x}_{\mathrm{m}}}>0\Rightarrow \frac{\partial {M}_n}{\partial {x}_{\mathrm{m}}}>0, $$

The above equations explain that when the multiplication layer thickness *x*_m_ is smaller than the critical point $\frac{-\alpha }{\partial \alpha /\partial {x}_{\mathrm{m}}}$, the breakdown voltage declines. When *x*_m_ is larger than that point, the breakdown voltage slightly rises. The value of the critical point calculated from the Eqs. (10)–(12) is ~ 0.2 μm, which is close to the simulated result in Fig. 5.

From the electric field distribution, with the increasing thickness of multiplication layer, the electric field in the absorption layer and the grading layer decreases, making the electrons more difficult to punch through the layers, so the punchthrough voltage increases. From the simulation results, in order to get a larger operating voltage range, the doping and thickness of the multiplication layer can be 4 × 10^16^ cm^−3^ and 0.05 μm, respectively.

**Fig. 6:**
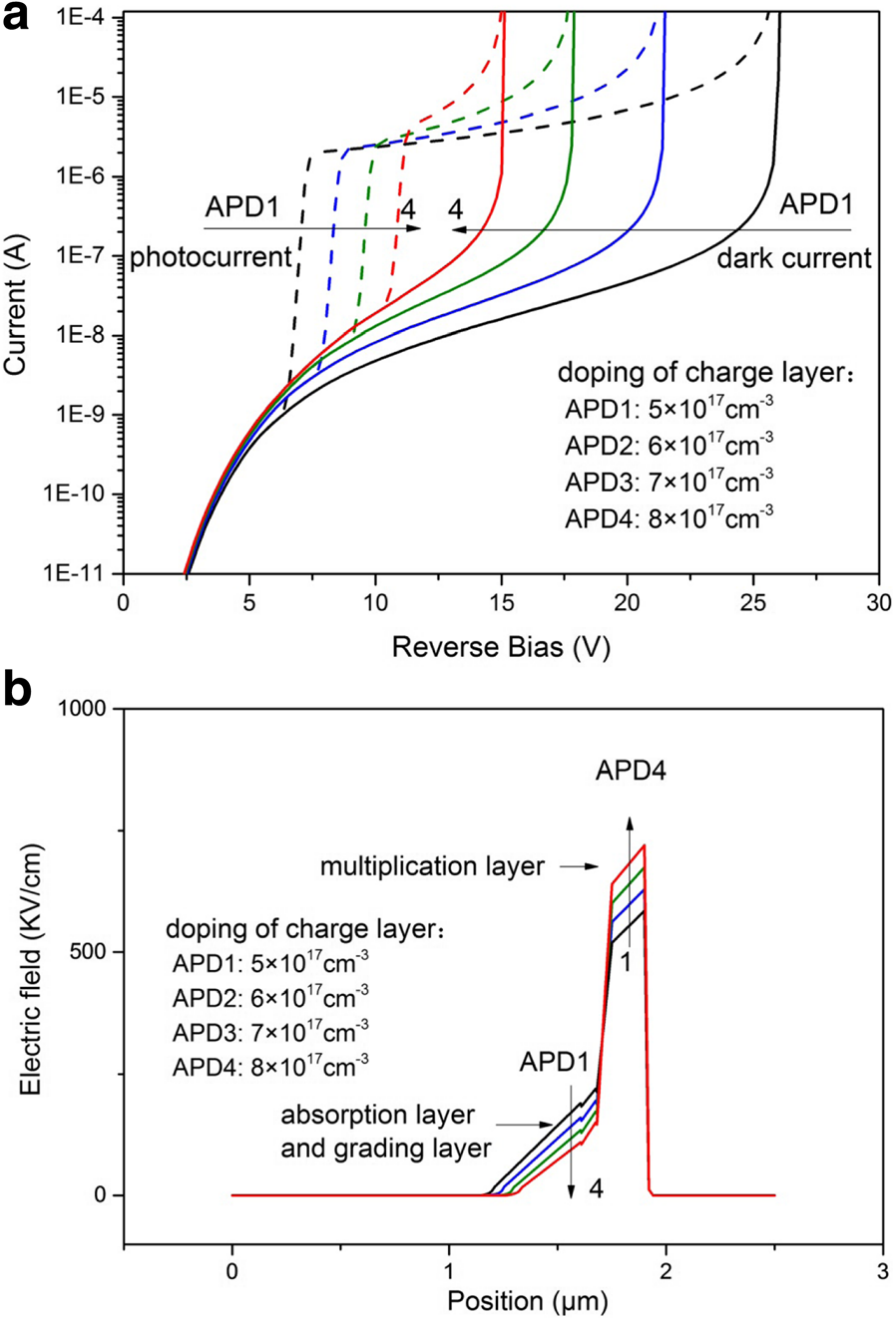
**a** Current–voltage characteristic of avalanche photodiode with different charge layer doping concentrations. **b** Distribution of electric field, biased at 15 V

**Fig. 7:**
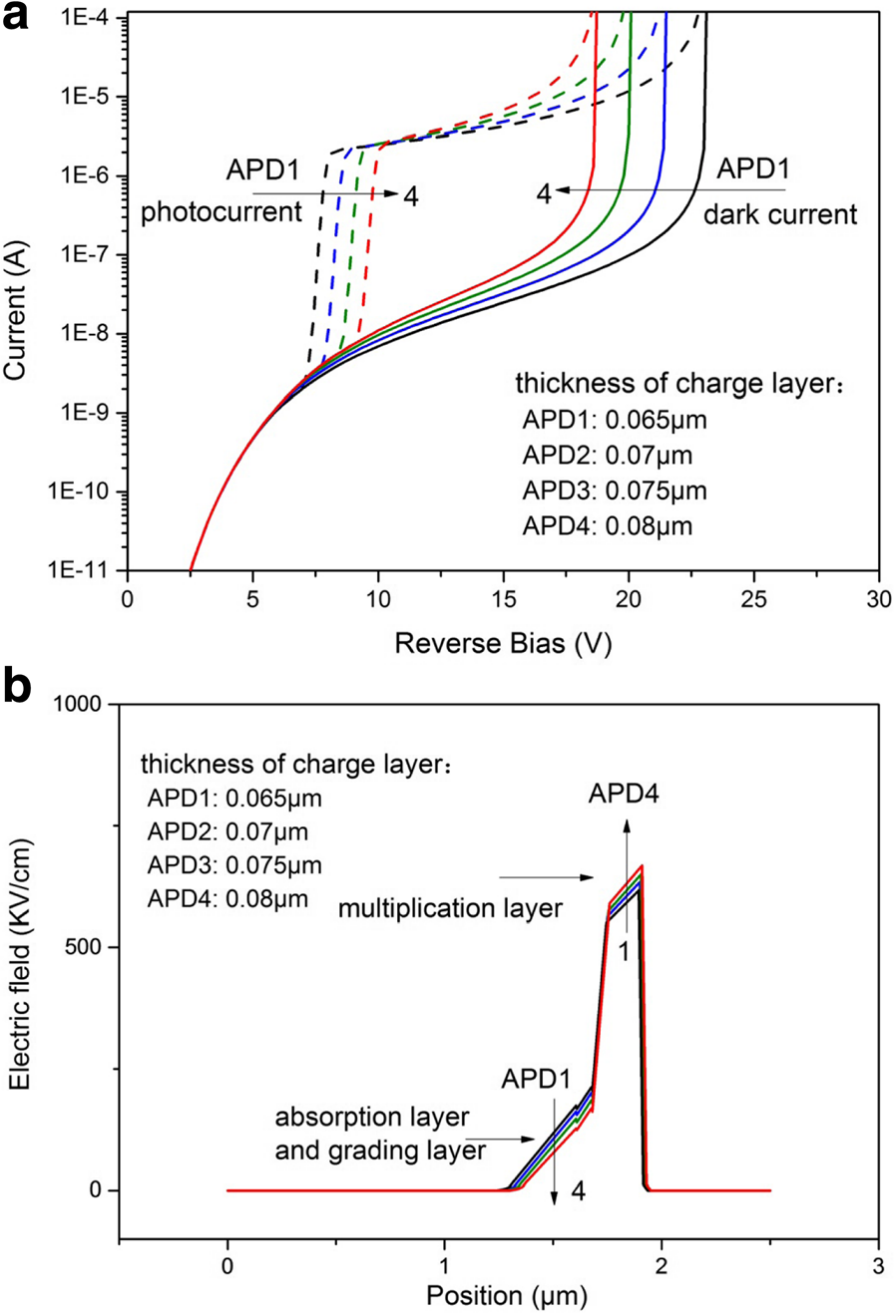
**a** Current–voltage characteristic of avalanche photodiode with different charge layer thicknesses. **b** Distribution of electric field, biased at 15 V

The electric field in the multiplication layer is enhanced by the charge layer to ensure the multiplication effect occurs in the multiplication layer. The thickness and the doping concentration of the charge layer can control the electric field in the multiplication layer. Figure 6 shows the dark and illuminated current characteristics with different doping concentrations (5 × 10^17^ ~ 8 × 10^17^ cm^−3^). With the increasing of doping concentration, the punchthrough voltage increases and the breakdown voltage decreases. Figure 7 shows the *I*–*V* characteristics with different thicknesses of the charge layer, and we can observe that with the increasing thickness, the punchthrough voltage increases while the breakdown voltage decreases [19, 20]. With the increasing thickness and the doping of charge layer, the electric field in the absorption layer and the grading layer decreases, and it makes the electron more difficult to punch through the layers, so the punchthrough voltage increases, but the electric field in multiplication layer increases with the increased thickness and the doping of charge layer. The thickness and doping concentration of the charge layer only affect the voltage distribution in the APD, so with the change of the parameters, the punchthrough voltage and the breakdown voltage change monotonously. Based on the simulation results, to further increase the operating voltage range, the doping and thickness of the charge layer can be 5 × 10^17^ cm^−3^ and 0.065 μm, respectively.

## Conclusions

In summary, we simulated and analyzed the punchthrough voltage and the breakdown voltage with the change of the parameters of the charge layer and multiplication layer. We found that with the increase of the thicknesses and the doping concentrations of the charge layer and the multiplication layer, the punchthrough voltage increases; with the increase of the doping concentrations of two layers and the thickness of the charge layer, the breakdown voltage decreases; with the increase of the thickness of the multiplication layer, the breakdown voltage first rapidly declines then slightly rises. Results show that the range of the operating voltage can be changed significantly by the charge layer and multiplication layer.

## Abbreviations

2D: Two-dimensional
FPA: Focal plane array
*I*–*V*: Current–voltage
SAGCM APDs: Separate absorption, grading, charge, and multiplication avalanche photodiodes
SAM APDs: Separate absorption and multiplication avalanche photodiodes
SRH: Shockley–Read–Hall
